# Effects of organic food consumption on human health; the jury is still out!

**DOI:** 10.1080/16546628.2017.1287333

**Published:** 2017-03-06

**Authors:** Marcin Barański, Leonidas Rempelos, Per Ole Iversen, Carlo Leifert

**Affiliations:** ^a^ Nafferton Ecological Farming Group (NEFG), School of Agriculture, Food and Rural Development, Newcastle University, Newcastle upon Tyne, UK; ^b^ Department of Nutrition, Institute of Basic Medical Sciences, University of Oslo, Oslo, Norway

**Keywords:** Organic farming, environment, sustainability, organic food nutritional composition, human health

## Abstract

The most recent systematic literature reviews and meta-analyses have indicated significant and nutritionally-relevant composition differences between organic and conventional foods. This included higher antioxidant, but lower cadmium and pesticide levels in organic crops, and higher omega-3 fatty acids concentrations in organic meat and dairy products. Also, results from a small number of human cohort studies indicate that there are positive associations between organic food consumption and reduced risk/incidence of certain acute diseases (e.g. pre-eclampsia, hypospadias) and obesity. Concerns about potential negative health impacts of organic food consumption (e.g. risks linked to lower iodine levels in organic milk) have also been raised, but are not currently supported by evidence from human cohort studies. However, there is virtually no published data from (1) long-term cohort studies focusing on chronic diseases (e.g. cardiovascular disease, diabetes, cancer, and neurodegenerative conditions) and (2) controlled human dietary intervention studies comparing effects of organic and conventional diets. It is therefore currently not possible to quantify to what extent organic food consumption may affect human health.

## Introduction

The demand for organic food has increased rapidly over the last 25 years in many developed countries in Europe, North America, and Asia/Oceania [1]. Demand is mainly driven by consumer perceptions that organic farming is more sustainable, and delivers environmental sustainability, biodiversity, animal welfare, and food quality and safety benefits compared to intensive conventional farming.

**Figure 1.**
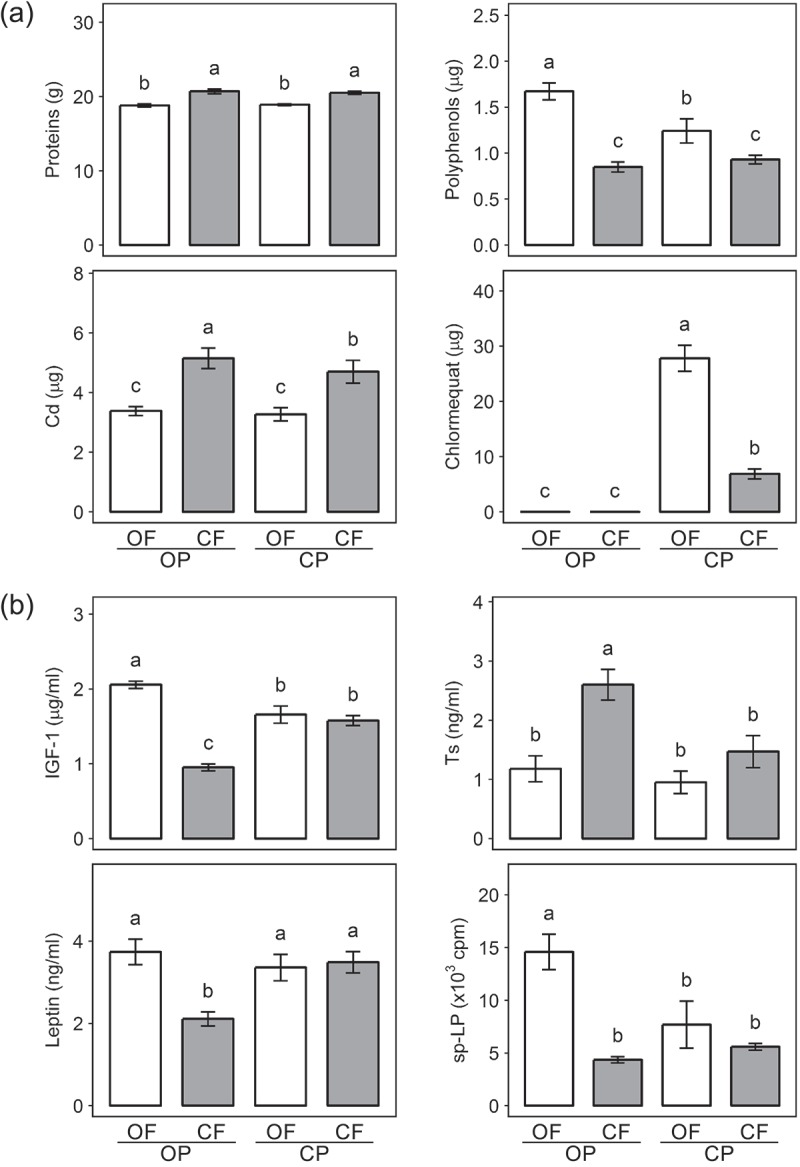
Effect of organic crop protection (OP) or conventional crop protection (CP), and organic fertility management (OF) or conventional fertility management (CF), on (1) the concentration of protein, polyphenols, cadmium, and chlormequat in 100 g of experimental animal feed, and (2) plasma insulin-like growth factor 1 (IGF-1), testosterone (Ts), leptin and spontaneous lymphocyte proliferation (sp-LP) in Wistar rats fed with these feeds. Results shown as means ±SEM of (1) n = 4 field replications, or (2) n = 24 animals; different letters above bars indicate significant difference (*P* < 0.05) determined by Tukey’s HSD test. Data from paper by Średnicka-Tober et al. [26], with the permission from authors.

While there is increasing scientific evidence for biodiversity and environmental sustainability-related benefits of organic farming [2–6], there is still considerable scientific controversy about whether or not, and to what extent organic production methods result in food quality and safety, and human health gains [7–11]. We therefore critically discuss the currently available evidence for composition differences and potential health impacts of organic food consumption below.

## Meta-analyses of composition differences

A series of recent systematic reviews and meta-analyses of published data have shown that there are significant differences in the concentrations of nutritionally relevant compounds between organically and conventionally produced foods [12–14]. Specifically, these systematic reviews reported that:

- organic crops have higher antioxidant activity and between 18 and 69% higher concentrations of a range of individual antioxidants; increased intakes of polyphenolics and antioxidants has been linked to a reduced risk of certain chronic diseases such as cardiovascular and neurodegenerative diseases and certain cancers (discussed by Barański et al. [12]);
- conventional crops have higher levels of the toxic metal cadmium, and are four-times more likely to contain detectable pesticide residues; there are general recommendations to minimise the intake of pesticides and cadmium to avoid potential negative health impacts (discussed by Barański et al. [12]);
- conventional crops also have higher concentrations of protein, nitrogen, nitrate, nitrite, respectively; increased intakes of these compounds have been linked to both positive and negative health impacts (discussed by Barański et al. [12]);
- organic meat, milk, and dairy products have approximately higher concentrations of nutritionally-desirable omega-3 fatty acids; intakes of very long chain omega-3 fatty acids in Western diets and there are EFSA (European Food Safety Authority) recommendation to at least double their intake (discussed by Średnicka-Tober et al. [13,14]);
- organic milk was reported to contain higher levels of total conjugated linoleic acid (CLA), higher iron and α-tocopherol concentrations, which are all considered to be nutritionally desirable, although the evidence for health benefits of CLA is mainly from in vitro and animal studies (discussed by Średnicka-Tober et al. [13]);
- conventional milk was estimated to have and higher concentrations of iodine and selenium, respectively; milk is not a major source for selenium, but may be the main source of iodine in countries were iodised salt in not widely available or used; there is concern that the lower iodine content in organic milk may cause iodine deficiency (especially during pregnancy and/or in individuals with low milk consumption) and associated negative health impacts (e.g. impaired foetal brain development) (discussed by Średnicka-Tober et al. [13]);
- conventional meat has slightly, but significantly higher concentrations of the saturated fatty acids myristic- and palmitic acid, which were linked to an increased risk of cardiovascular disease (discussed by Średnicka-Tober et al. [14]).

GRADE (grading of recommendations, assessments, development, and evaluation) assessment showed that the overall strength of evidence was good or moderate for many of the parameters listed above (e.g. total antioxidant activity, phenolic acids, flavonoids, flavanones, flavanols, anthocyanins stilbenes and nitrite in crops; total PUFA in milk and meat; n-3 PUFA, CLA, I, Fe, Se in milk), but low for others (e.g. certain individual or groups of antioxidants, cadmium, and nitrate in crops; α-tocopherol and carotenoids in milk, n-3 PUFA in meat) [12–14].

Also, for a range of parameters (especially in meat) for which significant differences were identified by meta-analyses GRADE assessment showed high inconsistency, poor precision and/or publication bias [12–14]. This indicates that for a range of parameters the currently available evidence base is still too small for accurate meta-analyses and/or that confounding factors (e.g. differences in agronomic and/or pedoclimatic conditions between countries in which studies were carried out) resulted in high variability (further limitations of the currently available evidence base for composition differences are described below).

Meta-analyses carried out prior to 2014 were all based on a smaller evidence base (number of publications/data), but produced broadly similar results, when they analysed the same parameters [7–11]. Brandt et al. [9] reported higher levels of antioxidants in organic crops. Smith-Spangler et al. [11] reported higher concentrations of phenolic compounds (the main group of antioxidants found in crop plants), risk of pesticide residues in organic crops, higher concentrations of omega-3 fatty acids in milk, and that the majority of published studies found higher cadmium concentrations in conventional crops. Palupi et al. [10] only reviewed studies on milk composition published between March 2008 and April 2011, and reported significantly higher concentrations of omega-3 fatty acids, CLA, and tocopherol in organic milk. Dangour et al. [15] (who pooled data for milk, meat, and eggs) found a trend towards higher concentrations of omega-3 polyunsaturated fatty acids (PUFA) in organic animal product, but did not include these results in the published paper [8]. Based on these results it is tempting to conclude that (except for iodine intake with milk) organic food consumption results in higher dietary intakes of a range of nutritionally desirable compounds such as antioxidants, certain vitamins, and omega-3 fatty acids, but lower intakes of nutritionally undesirable pesticides, Cd, and saturated fatty acids.

However, it is virtually impossible to accurately estimate change in dietary intakes, since there are still substantial gaps of knowledge with respect to composition differences between organically and conventionally produced foods. For example, there is (1) a need to systematically review mycotoxin levels in crops (especially cereals) and/or composition differences in eggs from organic and conventional farms, (2) insufficient data to accurately estimate the magnitude of differences for individual crops and meat products from different livestock species, which means it is currently not possibly to accurately estimate differences in dietary intakes of the most desirable and undesirable compounds from organic and conventional food based diets, and (3) not enough published information to compare concentrations of a wide range of nutritionally relevant nutrients (e.g. water soluble vitamins, and many minerals in milk and meat) and undesirable compounds (e.g. pesticides, antibiotics, hormones, synthetic food additives in milk and meat) in a meta-analysis [12–14]. Also, a range of methodological issues related to systematic reviews of composition data have been raised and need to be resolved [16].

### Human cohort studies

A small number of human cohort studies and animal dietary intervention studies have identified associations between organic food consumption and specific health, and health-related physiological parameters. Most human cohort studies were mother-and-child dyad cohorts and reported positive associations between organic vegetable and/or dairy consumption and risks of (1) pre-eclampsia in mothers [17], (2) hypospadias in baby boys [18,19], and/or (3) eczema in infants [20].

A sub-study (of about 54 000 adults) of the French-Belgium Nutrienet-Sainté cohort reported that regular consumers of organic food had a substantially lower risk of being overweight or obese [21]. The association between organic food consumption and reduced risk overweight/obesity was also found when data were adjusted for age, physical activity, education, smoking status, energy intake, restrictive diet, and adherence to public nutritional guidelines. In the paper the authors state that these data must be interpreted with caution since the study has several limitations.

Also, a subgroup of a large UK cohort study focused on cancer incidence in middle-aged women showed that there is a weak association between organic food consumption and a reduced incidence of non-Hodgkin’s lymphoma, although the study was based on an observation period of only seven years [22].

However, there are a range of confounding factors that may have influenced the outcome of all cohort studies since organic and conventional consumers are known to differ in a range of other lifestyle factors (e.g. diet composition, use of medicines, health supplements and vaccinations, and/or levels of exercise, alcohol consumption, and smoking) which are often difficult to properly factor out in cohort studies [16,23,24].

### Animal dietary intervention studies

There are also a small number of published animal dietary intervention studies in which the effects of organic vs. conventional food consumption were compared (reviewed by Velimirov et al. [25] and Średnicka-Tober et al. [26]). All studies identified significant effects on animal growth and/or physiological parameters (e.g. body composition, plasma antioxidant and hormone levels, immunoglobulin concentrations and/or immune system responsiveness) of switching from conventional to organically produced feed (Figure 1). However, the parameters assessed and analytical methods, animal species and/or experimental designs used differed between studies, which make it difficult to identify consistent trends across studies (reviewed by Velimirov et al. [25] and Średnicka-Tober et al. [26]).

## Knowledge gaps and future research needs

It is increasingly accepted that there can be nutritionally relevant composition differences between organic and conventional foods and there is some evidence for potential benefits of organic food consumption from human cohort studies. However, considerable uncertainty/controversy remains on whether or to what extent these composition differences affect human health.

To overcome this uncertainty it is essential to (1) address a range of methodological issues in both the available meta-analyses of composition data and dietary cohort studies [16,23,24], (2) carry out additional well-designed food composition comparisons for specific crops and meat types to allow reliable comparisons of dietary intakes of nutritionally relevant compounds with organic and conventional foods [13,14], (3) carry out well-designed human dietary intervention studies comparing the effect of organic vs. conventional food consumption on health and health-related physiological parameters [12–14,23]. Also, studies that allow a more mechanistic understanding of how organic food consumption does affect health are required. This could, for example, be based on dietary intervention studies with animal models prone to specific diseases.
